# The claustrum’s proposed role in consciousness is supported by the effect and target localization of *Salvia divinorum*

**DOI:** 10.3389/fnint.2014.00020

**Published:** 2014-02-26

**Authors:** Klaus M. Stiefel, Alistair Merrifield, Alex O. Holcombe

**Affiliations:** ^1^The MARCS Institute, University of Western SydneySydney, NSW, Australia; ^2^NPS MedicinewiseSydney, NSW, Australia; ^3^School of Psychology, University of SydneySydney, NSW, Australia

**Keywords:** claustrum, consciousness, *Salvia divinorum*, salvinorin A, κ-opioid receptor

## Abstract

This article brings together three findings and ideas relevant for the understanding of human consciousness: (I) Crick’s and Koch’s theory that the claustrum is a “conductor of consciousness” crucial for subjective conscious experience. (II) Subjective reports of the consciousness-altering effects the plant *Salvia divinorum*, whose primary active ingredient is salvinorin A, a κ-opioid receptor agonist. (III) The high density of κ-opioid receptors in the claustrum. Fact III suggests that the consciousness-altering effects of *S. divinorum*/salvinorin A (II) are due to a κ-opioid receptor mediated inhibition of primarily the claustrum and, additionally, the deep layers of the cortex, mainly in prefrontal areas. Consistent with Crick and Koch’s theory that the claustrum plays a key role in consciousness (I), the subjective effects of *S. divinorum* indicate that salvia disrupts certain facets of consciousness much more than the largely serotonergic hallucinogen lysergic acid diethylamide (LSD). Based on this data and on the relevant literature, we suggest that the claustrum does indeed serve as a conductor for certain aspects of higher-order integration of brain activity, while integration of auditory and visual signals relies more on coordination by other areas including parietal cortex and the pulvinar.

## CRICK AND KOCH’S IDEAS ON THE ROLE OF THE CLAUSTRUM

The late Francis Crick proposed that at any one moment, human subjective consciousness of perceptual contents^1^ is brought about by the activity of a limited number (~10^5^) of neurons (Crick, 1995; Crick and Koch, 2003). According to Crick’s analysis, these neurons must: (1) Be central in the connection scheme of the human brain, not too close to primary sensory or motor areas. (2) Involve a number of sensory areas, since consciousness integrates several sensory modalities. (3) Have activity correlated with conscious experience, even in situations where it is dissociated from direct sensory input (for instance during the perception of visual illusions). Importantly, the identity of these neural populations will likely change as the contents of conscious experience change. Crick and other authors have suggested that some brain region must act as a “conductor” of this dynamic “conscious field” (Tononi and Edelman, 1998a)^2^, “dynamical core” (Tononi and Edelman, 1998b; Dehaene and Changeux, 2004) or “neuronal workspace” (Crick and Koch, 2005).

**FIGURE 1:**
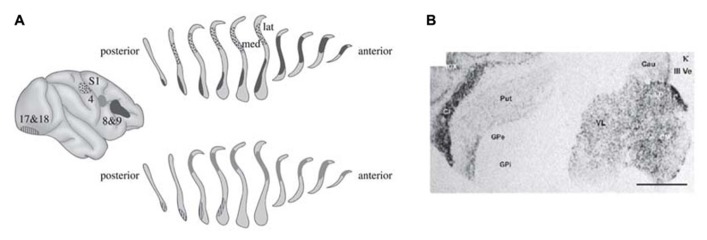
**Connectivity and κ-opioid receptor density of the claustrum in the human brain. (A)** The claustrum is strongly connected to diverse cortical areas, with the sections of the claustrum receiving cortical inputs overlapping. This anatomical connection pattern, amongst other things, leads Crick and Koch to propose that the claustrum acts as a director of consciousness. The connectivity was established by injecting horseradish peroxidase into the cortex, and subsequently tracing the marked cells in the claustrum. Figure from Crick and Koch (2005), as modified from the original study (Phelps and LeDoux, 2005). **(B)** In situ hybridization signal of κ-opioid receptor mRNA. The darker the staining of the tissue, the higher the density of κ-opioid receptor mRNA, and subsequently of κ-opioid receptors. Cl: claustrum. Scale bar = 5 mm. Figure from Peckys and Landwehrmeyer (1999).

In the last paper Crick authored before his death, he and Koch argued that the claustrum is an ideal candidate for this role (Crick and Koch, 2005). The claustrum is a brain region located between the insular cortex, piriform cortex and the caudate-putamen (Franklin and Paxinos, 2007), see **Figure 1**. It is highly connected to a number of cortical areas in a mostly reciprocal manner (Carman et al., 1964; Shameem et al., 1984; Neal et al., 1986; Sadowski et al., 1997). This strong and complex interconnectivity with the cortex makes it a prime candidate for the role of the director of the conscious field.

Crick and Koch’s hypothesis about the central role of the claustrum in consciousness has received some attention (Smythies et al., 2012, 2013, 2014) but unfortunately the evidence has been limited to anatomy and behavioral effects on animals other than primates. Here we argue that because the psychoactive plant *Salvia divinorum* has its effect primarily on neuromodulator receptors concentrated in the claustrum, analysis of the subjective effects caused by *S. divinorum’s* active compound provide a novel source of evidence regarding the role of the claustrum in humans.

This new evidence essentially supports Crick and Koch’s arguments from anatomy. A detailed look at the subjective effects together with a review of some of the literature leads us to extend Crick and Koch’s theory of the claustrum’s role in human consciousness. Specifically, we suggest that the claustrum is one of several brain-wide integrators, together with the parietal cortex and the pulvinar (the role of the pulvinar is already discussed in Smythies et al., 2013). We discuss these theoretical proposals, and suggest specific tests using neuroimaging and neural recordings in conjunction with administration of *Salvia*.

## THE PSYCHOACTIVE COMPOUND OF *S. divinorum* AND THE κ-OPIOID RECEPTORS OF THE BRAIN

The *S. divinorum *plant, of the* Lamiaceae *mint family, is native to the Oaxaca region in southern Mexico and is traditionally orally ingested by Mazatecs as an inebriate in religious and spiritual contexts (Siebert, 1994). Its main active compound, salvinorin A, has a threshold effective dose of 250 μg to evoke mind-altering effects in an average-sized adult (humans: Siebert, 1994; rodents: Ansonoff et al., 2006).

Salvinorin A is a κ-opioid receptor agonist (Cooper et al., 2002; Roth et al., 2002; Chavkin et al., 2004). Opioid receptors are a class of neuromodulators-receptor proteins embedded in the neural membrane. They convey a signal from the outside of the neuron, the binding of an opioid molecule, to the inside of the neuron, by releasing signaling molecules (2nd messengers) into the cytoplasm. Four classes (with several subtypes) of such receptors, δ, κ, μ, and nociception receptors exist, each with different agonist specificities and signal transduction mechanisms. In the human brain, a number of endogenous agonists activate these receptors. Smoking or orally ingesting *S. divinorum* is also believed to activate them.

An intracellular IP_3_ and cAMP^3^ based 2nd messenger cascade (Law et al., 2000) elicited by the κ-opioid receptors yields downstream cellular effects (Henry et al., 1995). Cellular excitability decreases via an increase of the inward rectifier potassium currents (Tallent et al., 1994). Additionally, κ-opioid receptors down-regulate N-type calcium currents, which, via the reduction of presynaptic Ca^2^^+^ influx, likely leads to a reduction of excitatory and inhibitory neurotransmitter release. The effects of κ-opioid receptors are thus inhibitory, both by reducing the amount of input a neuron is receiving and by reducing the response to that input.

The distributions of neurotransmitter κ-opioid receptors in brains have been measured both by detecting the density of receptor mRNAs and by detecting the receptor-mediated metabolism of radioactively labeled 2nd messenger precursors. In human brains, κ-opioid receptor expression was measured by mRNA *in situ* hybridization (Peckys and Landwehrmeyer, 1999). High densities were found in the striatum, hippocampal dentate gyrus, deep cortical layers (V and VI, with more expression in the prefrontal than in the occipital cortex) and, especially, in the claustrum. The claustrum showed the strongest signal, in fact it was the only brain region in which nearly all cells were labeled with dense to very dense labeling density (**Table 1**).

**Table 1:** κ-opiod receptor densities in the human brain.

Brain region/layer	Density of labeled neurons	Grain density per labeled neuron
**Prefrontal cortex**
Layer I	0	0
Layer II	++	+ to ++
Layer III	+ to ++	+ to ++
Layer IV	0	0
Layer V	+++	++ to +++
Layer VI	+++	++
**Primary visual cortex**
Layer I	0	0
Layer II	+	+ to ++
Layer III	+	+
Layer IV	0	0
Layer V	++ to +++	+ to ++
Layer VI	++	+
**Hippocampus**
Dentate gyrus	+++	++
CA1	+	++
CA2	+	++
CA3	++	+++
CA4	+	++
**Striatal region**
Accumbens nucleus	+++	++
Putamen anterior part	+++	+++
Putamen posterior part	++	+ to ++
Caudate nucleus anterior	+++	+ to ++
Caudate nucleus posterior	++	+ to ++
Ventral pallidum	++	+
Globus pallidus external	0	0
Globus pallidus internal	0	0
**Claustrum**	++++	+++ to ++++

*Adapted from Peckys and Landwehrmeyer (1999). 0, no signal; + to +++, increasing receptor densities.*

In the macaque monkey brain, κ-opioid receptor activity was measured by monitoring the agonist-induced binding of a radioactively labeled GTP-analog ([^35^S]GTPγS)^4^. Strong activity was found in the limbic and association cortex, ventral striatum, caudate, putamen, globus pallidus, claustrum, amygdala, hypothalamus, and substantia nigra (Sim-Selley et al., 1999). The authors report that “A very high level of κ_1_-stimulated [^35^S]GTPγS binding was observed in the claustrum, with an area of especially high stimulation in the ventral claustrum, adjacent to the amygdala”. While there was evidence for κ-opioid receptor activity in other brain regions as well, the densities were markedly higher in the aforementioned regions.

The unusually high κ-opioid receptor density in the claustrum makes it a particularly good candidate area for the consciousness altering effects of salvinorin A. Compared to other brain regions, this high receptor density will likely lead to an onset of inhibition of activity in the claustrum at lower concentrations, and to a stronger inhibition at equal concentrations of salvinorin A.

While the receptor density already strongly suggests that the consciousness-altering effects of *Salvia* are related to claustrum disruption, we should also consider the other parts of the brain with significant κ-opioid receptor densities. The striatum, caudate, putamen, substantia nigra, and globus pallidus are commonly considered to be part of an integrated system involved in action selection, reinforcement learning, and motor control, and are not likely neural correlates of consciousness (Wilson, 2004). The hypothalamus is considered to be responsible for the regulation of metabolic processes as part of the autonomous nervous system. It is an unlikely candidate for a role in consciousness other than creating certain states of arousal necessary for consciousness. The amygdala is a brain region thought to be involved in emotional processing, such as fear and fear conditioning (Phelps and LeDoux, 2005). Some subjects report a component of fear in their *S. divinorum *evoked experiences. However, this effect is distinct from the consciousness-altering effects we are discussing here. This leaves us with the deep layers of the frontal and prefrontal cortex and the claustrum as relevant *S. divinorum/*salvinorin A target areas. Arguments both from receptor densities, as well as from exclusion of brain regions due to known functions point in their direction. Most likely, these are the brain areas which, when inhibited by salvinorin A, give rise to the intense consciousness-altering experiences reported by users of *S. divinorum*. The frontal and prefrontal cortex are known as areas involved in planning, higher-order executive and social functions (Cicerone and Tanenbaum, 1997; Beer et al., 2006). Two recent studies have also found dopaminergic activity of salvinorin A (Grundmann et al., 2007; Listos et al., 2011), which could have additional influence on the frontal cortex. A disruption of this area could explain some effects of *S. divinorum* (see below); however the proposed roles of the claustrum are sufficiently different from the proposed roles of the frontal and prefrontal cortex to allow some distinction in the analysis of the subjective effects.****

## CONSCIOUSNESS-ALTERING EFFECTS OF SALVINORIN A

Unfortunately for scientists, human consciousness is not accessible to outside observers. Observers’ reports about their consciousness can be unreliable, but such subjective reports are the only source of information about a conscious experience, and are therefore valuable for understanding consciousness.

For *S. divinorum*, subjective reports indicate marked differences between the experiences associated with it versus those of other psychoactive drugs. Baggott et al. (2010) conducted an online survey of *Salvia* users, asking them to compare it to other methods of altering consciousness. The most frequent (38%) response was that it is unique. Experiences with Salvia are sometimes likened to lucid dreams and are usually described as highly interesting but frequently also as terrifying and unpleasant.

In the discussion below, the effects of *Salvia* described are based on anecdotal reports together with a quantitative analysis of subjective reports obtained from Erowid.org (http://www.erowid.org/). Erowid.org is a curated website with tens of thousands of informational documents about drugs. Members of the public post accounts of their experiences after ingesting various substances, and the administrators of the site sometimes work with researchers (Baggott et al., 2010). We (independently of the administrators of the site) initially chose 63 *Salvia* experiences and 63 lysergic acid diethylamide (LSD) experiences randomly from the site. We are well aware of the limitations of these reports, including a lack of control over the dosage and no prior screening of participants. However, there is on average no reason to doubt the sincerity of these reports, and they constitute a large dataset of human experiments with psychoactive substances that are controlled in many countries. Because the effect of *S. divinorum* may result from a somewhat specific disruption of the activity of the claustrum, the reports are valuable subjective descriptions of the effects of such a disruption.

**FIGURE 2:**
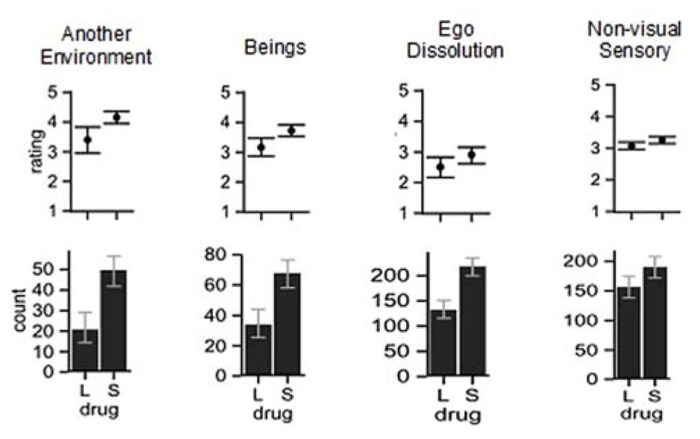
**Comparison of the average rating (top row) and number of trip questions that received ratings (bottom row) for *S. divinorum* and LSD trip reports.**> See trip report analysis deposited at http://dx.doi.org/10.6084/m9.figshare.902215 for associated statistics. Error bars are 95% confidence intervals. For the counts, the confidence intervals were calculated with Agresti and Caffo’s (2000) add-4 method and for the mean ratings, they were bootstrapped.

Our exploratory analysis of the trip reports, the methodology and results of which is described in detail at http://dx.doi.org/10.6084/m9.figshare.902215, used questions from inventories developed to assess the effects of psychoactive drugs (Dittrich, 1985; Strassman et al., 1994; Studerus et al., 2010), together with some novel questions relevant to Crick and Koch’s conductor of consciousness theory. One student and one postdoctoral scholar of psychopharmacology discussed with us the questions before reading all the trip reports and scoring them with the questions. We were interested in whether certain aspects of experience were disrupted, and grouped the questions into several categories. Inferential statistics suggest that Salvia experiences differed from LSD experiences on at least four categories, which are depicted in **Figure 2**. The four categories of questions that yielded significantly higher scores with Salvia are “ego dissolution” (a concept used by Dittrich, 1985 for what became the ASC questionnaire), “another environment,” “beings,” and “nonvisual sensory.”

Regarding Salvia’s particularly strong effect on the “another environment” category, the high ratings reflect frequent loss of awareness of the subjects’ current surroundings, replaced by the experience of being in a completely different location. The different location sometimes is a real place, possibly visited decades earlier, and in other cases completely imaginary. One subject reported that he “all of a sudden was in my childhood bedroom as it looked twenty years ago”. Another subject reported being in a scene that “almost alluded to an African or Haitian village” (Arthur, 2008). Disturbances of consciousness of that kind seems consistent with Crick and Koch’s theory of the claustrum as a conductor of consciousness, as experience of presence in a certain locality can reasonably be interpreted as a synthesis of a number of qualitatively different contents of consciousness. But while Crick and Koch wrote of disturbances to the synthesis specifically of sensations associated with a perceived object, these other-location experiences suggest wholesale substitution of current sensory input with other sensations, sometimes constructed from memories, often forming a new coherent whole entirely distinct from the current sensory environment.

A second prominent feature of *S. divinorum* experiences is its stronger effects on the “beings” questions. These correspond to the subjective presence of non-existent people or sentient beings. One subject reported, for example “The Salvia spirits (so I believed at the time) begun pulling me with fuzzy green arms covered in eyes to take me into their dimension. Their vague form was a green loop functioning as both head and arms, with a translucent body in between. They were playful, unthreatening yet determined…The most powerful part of the hallucination was my belief that the spirits were real” (erowid.org/exp/72338) While there are also encounters with beings in hallucinations induced by serotonergic psychedelics, they appear to be less common and our data supports this for the case of LSD. Of the trip reports analyzed, 31 of the *Salvia* reports mentioned interactions with other beings, against 18 of the LSD reports. Also, experiencing actual conversations with such beings seems to be rare with other drugs, for which beings appear as visual hallucinations, but are rarely perceived to utter speech. One possibility is that these effects are caused by disruption of self image, as discussed below. Another possibility is that the aforementioned physiological effects of salvinorin A on the social regions in the frontal and prefrontal cortical areas are responsible for the experiences of beings.

A third feature of the influence of *S. divinorum* is severely altered body image. Subjects sometimes report perceiving their bodies as geometric objects, losing any feeling for the existence of their bodies whatsoever, and sometimes existing entirely non-spatially or outside of time. One subject stated: “It’s as if I have no body, but I can still feel that I’m connected by the roof of my mouth to this slab thing. It’s completely absurd. Then I start to feel like the slab is being held up vertically by somebody. I can’t see him, but it’s as if he has been carrying the slab around for eternity, and that I have always been here attached to it.”****(erowid.org/exp/78727).****Several component questions contributed to the significantly higher ratings for the associated “ego dissolution” category for the Salvia trip reports. These included “I was not able to complete a thought, my thought repeatedly became disconnected,” “It seemed to me as though I did not have a body anymore,” “Thoughts of present or recent past personal life,” and “Feel removed, detached, separated from body.” While alterations of representation of the self and body also occur under the influence of serotonergic psychedelics (including, in our dataset, LSD), those changes may be less extreme. It seems that under serotonergic psychedelics, the proportions and sizes of body parts are altered, but the modified percepts still originate from the subject’s current sensory input. In contrast, during *S. divinorum* experiences, the origin of body experiences is completely altered.

Substantial progress has been made recently in understanding the neural basis of body image and perspective. Imaging of functioning human brains, in conjunction with visuo-tactile stimulation situations that elicit out-of-body experiences and lesion studies point to the temporo-parietal cortical junction as an area that encodes location of the self (Ionta et al., 2011). It has been suggested that the same function of encoding self location may, when disrupted, result in the experience of other beings. In a woman undergoing surgery for epilepsy, stimulation of the left temporo-parietal junction yielded the feeling of a shadowy person immediately behind her that mimicked her posture and actions (Arzy et al., 2006). While the patient interpreted the figment as being not herself, the researchers suggest that it was a duplicated and displaced self body image, interpreted as another being. With *Salvia* however typically it does not appear that the other being mimics the subject’s movements (or even intended movements). While the temporoparietal cortex did not show a strong signal in the κ-opiate receptor localization studies discussed above, connections between the claustrum and the parietal cortex do exist (Baizer et al., 1993). We suggest that this indirect effect onto the parietal cortical areas is most likely responsible for the effects of *Salvia* on body image.

A fourth aspect of experience for which *Salvia* scored significantly higher was the “non-visual sensory” category, with the difference mainly driven by the following questionnaire items: “Gravity was in the wrong direction, or wrong force field,” “Feel as moving/falling/flying through space,” as well as the non-specific “Body feels different”. We suspect that the response to the former two questions, and possibly the last question, is related to the ego dissolution and body image disruption that occurs with *Salvia*. Recent work on neurological patients with impaired perception of the direction of gravity has emphasized that somatosensory information is very important, rather than solely vestibular signals (Barra et al., 2010). Blanke and Metzinger (2009) have linked disruption of somatosensory, vestibular, and gravity percepts to abnormal experiences of phenomenal selfhood. Somatosensory processing is also integral to body image, which as discussed above is highly disrupted in *Salvia* experiences. An alternative interpretation for the experiences in the “non-visual sensory” category is more in accord with Crick and Koch’s conductor hypothesis – a lack of a proper focus of consciousness aimed at the sensory inputs coming from the present surroundings. This interpretation would implicate the claustrum in a different way than body image distortion to bring about the non-visual sensory effects. In summary, the non-visual sensory effect (which statistically was weaker than the other categories) might reflect the same processes that disrupt body image, and/or substitution of current sensory inputs and representations with others unrelated to the immediate environment.

A recently published study reports that *S. divinorum *evokes synesthesia (Luke and Terhune, 2013). In our sample, both LSD and *S. divinorum* users reported synesthesia.

In sum, certain effects of *Salvia* consistent with claustral function disruption and Crick and Koch’s theory of the claustrum as a conductor of consciousness (effects on “another environment” and possibly “non-visual sensory”). Another class of Salvia effects was found that are at most indirect effects of claustral inactivation (“body image”), and effects likely brought about by an influence of Salvia on other brain areas (“beings”).

## SUMMARY AND DISCUSSION

Crick and Koch (2005) highlighted the mystery of the claustrum, and provided a stimulating theory of the claustrum’s possible role in consciousness. Here, consideration of the high density of κ-opioid receptors in the claustrum sparked the realization that insights into the claustrum’s role in consciousness might be gained by assessing the effects of *S. divinorum*/salvinorin A on humans. Using *Salvia* profoundly disturbs subjective experience, supporting the idea of Crick and Koch that the claustrum is important for consciousness. The analysis of the subjective reports presented here is in agreement with several studies which report significant hallucinogenic effects of *S. divinorum*, and effects different from those of serotroninergic hallucinogens (Johnson et al., 2011; Addy, 2012; Ranganathan et al., 2012; MacLean et al., 2013). The fact that besides the differences a considerable overlap exists between the effects of different hallucinogens is, in our opinion, evidence of the complexity and unified nature of the human psyche.

In principle, the analysis of the *Salvia*-mediated effects (likely affecting the claustrum) agrees with the role of the claustrum as a large-scale integrator or “conductor” of numerous far-flung cortical regions. However, our analysis of subjective reports of the effect of *Salvia* suggests that the claustrum is not the sole brain area concerned with across-modality or within-modality binding (see also Smythies et al., 2013).

Relative to LSD, *Salvia* was more likely to give users the impression of being in hallucinated locations with hallucinated beings, while severely distorting or disrupting their representation of their own body, sometimes with the experience of a more abstract, less physical existence. This is also characteristic of rapid-eye-movement sleep, during dreams. In both cases, conscious experience is decoupled from the signals being provided by the senses, with the brain given free rein to concoct novel scenarios, presumably based on recombination of previous experiences. Recent neuroimaging work has revealed brain areas whose activity appears to both correlated with and causally involved in the experience of scenes (the occipital place area and parahippocampal place area, Epstein and Kanwisher, 1998; Dilks et al., 2013), and future investigation should examine the involvement of these areas. A possible interpretation of these effects would be that the constructivist nature of perception is even more pronounced than in a sober state, with *Salvia* disrupting the proper coordination of sensory input and memories.

*Salvia* also disrupted the user’s representation of his body and self. Veridical representation of the self, like audiovisual binding, requires the integration of distinct cortical areas. Specifically, the representation of body image appears to involve somatosensory, vestibular, proprioceptive, and visual signals (Barra et al., 2010). The maintenance of body image may therefore be particularly dependent on simultaneous integration of multiple cortical areas. This is consistent with Crick and Koch’s likening of the claustrum to a symphony conductor.

The analysis of the subjective experiences caused by *Salvia* also suggests that the claustrum is involved in coordinating some brain areas, but not critical for (though possibly involved in) the binding of auditory and visual signals or color and motion (Stiefel and Holcombe, 2014) . If so, then what areas of the brain do mainly serve these functions? Neuropsychological evidence published after Crick and Koch’s paper have implicated another subcortical area, the pulvinar, in perceptual binding (Ward and Arend, 2007; Arend et al., 2008) along with the already-known importance of parietal cortex (Cohen and Rafal, 1991; Friedman-Hill et al., 1995).

Functional brain imaging of humans under the influence of *S. divinorum* should improve our understanding of the role of the claustrum. The fast pharmacokinetics of salvinorin A (onset in seconds, duration of minutes) are advantageous for electroencephalography (EEG) and functional magnetic resonance (fMRI) imaging studies in humans, as well as more invasive techniques in other animals. If**salvinorin A indeed inhibits the claustrum and disrupts large-scale cortical coordination, these proposed studies should yield a massive reorganization of cortical activity. It is impossible to predict the exact nature of the reorganization, since a specific theory of cortico-claustral interaction does not yet exist, but we speculate that one result will be decorrelation of the activity in somatosensory, vestibular, and other cortical areas.

## AUTHOR CONTRIBUTIONS

Klaus M. Stiefel, conceived the approach and researched the literature. Klaus M. Stiefel and Alex O. Holcombe, wrote the manuscript, designed, and directed the coding of the trip reports. Alex O. Holcombe and Alistair Merrifield performed preliminary analysis. Alex O. Holcombe, conducted the final data analysis and created the plots..

## Conflict of Interest Statement

The authors declare that the research was conducted in the absence of any commercial or financial relationships that could be construed as a potential conflict of interest.

## Footnotes

1 Crick’s definition of consciousness (Crick, 1995) is used here.

2 Searle’s nomenclature for this concept is used, without hyphens, in the remainder of the paper.

3 Inositol triphosphate and cyclic adenosine monophosphate.

4 Guanosine triphosphate, another molecule involved in signaling by κ$-opioid receptors.
